# Fructose and Sucrose Intake Increase Exogenous Carbohydrate Oxidation during Exercise

**DOI:** 10.3390/nu9020167

**Published:** 2017-02-20

**Authors:** Jorn Trommelen, Cas J. Fuchs, Milou Beelen, Kaatje Lenaerts, Asker E. Jeukendrup, Naomi M. Cermak, Luc J. C. van Loon

**Affiliations:** 1 NUTRIM School of Nutrition and Translational Research in Metabolism, Maastricht University Medical Centre, P.O. Box 616, 6200 MD Maastricht, The Netherlands; jorn.trommelen@maastrichtuniversity.com (J.T.); cas.fuchs@maastrichtuniversity.nl (C.J.F.); milou.beelen@maastrichtuniversity.nl (M.B.); kaatje.lenaerts@maastrichtuniversity.nl (K.L.); naomi.cermak1234@gmail.com (N.M.C.); 2 School of Sport, Exercise and Health Sciences, Loughborough University, Loughborough LE11 3TU, UK; asker@mysportscience.com

**Keywords:** substrate utilization, stable isotopes, metabolism, sugar

## Abstract

Peak exogenous carbohydrate oxidation rates typically reach ~1 g·min^−1^ during exercise when ample glucose or glucose polymers are ingested. Fructose co-ingestion has been shown to further increase exogenous carbohydrate oxidation rates. The purpose of this study was to assess the impact of fructose co-ingestion provided either as a monosaccharide or as part of the disaccharide sucrose on exogenous carbohydrate oxidation rates during prolonged exercise in trained cyclists. Ten trained male cyclists (VO_2_peak: 65 ± 2 mL·kg^−1^·min^−1^) cycled on four different occasions for 180 min at 50% W_max_ during which they consumed a carbohydrate solution providing 1.8 g·min^−1^ of glucose (GLU), 1.2 g·min^−1^ glucose + 0.6 g·min^−1^ fructose (GLU + FRU), 0.6 g·min^−1^ glucose + 1.2 g·min^−1^ sucrose (GLU + SUC), or water (WAT). Peak exogenous carbohydrate oxidation rates did not differ between GLU + FRU and GLU + SUC (1.40 ± 0.06 vs. 1.29 ± 0.07 g·min^−1^, respectively, *p* = 0.999), but were 46% ± 8% higher when compared to GLU (0.96 ± 0.06 g·min^−1^: *p* < 0.05). In line, exogenous carbohydrate oxidation rates during the latter 120 min of exercise were 46% ± 8% higher in GLU + FRU or GLU + SUC compared with GLU (1.19 ± 0.12, 1.13 ± 0.21, and 0.82 ± 0.16 g·min^−1^, respectively, *p* < 0.05). We conclude that fructose co-ingestion (0.6 g·min^−1^) with glucose (1.2 g·min^−1^) provided either as a monosaccharide or as sucrose strongly increases exogenous carbohydrate oxidation rates during prolonged exercise in trained cyclists.

## 1. Introduction

It has been well established that carbohydrate ingestion during prolonged moderate- to high-intensity endurance-type exercise increases exercise capacity and performance [1,2,3]. The observed improvements in performance with carbohydrate ingestion have been attributed to maintenance of plasma glucose concentrations and high rates of carbohydrate oxidation during the latter stages of exercise [1,4].

Glucose ingestion during exercise results in a maximal exogenous carbohydrate oxidation rate of ~1 g·min^−1^ [5,6]. The rate of exogenous glucose oxidation appears limited by intestinal glucose absorption [5,7]. The intestinal sodium-dependent glucose transporter 1 (SGLT1) may become saturated when large amounts of glucose or glucose polymers are ingested [7,8]. Interestingly, the intestine contains a distinct class of carbohydrate transporters, glucose transporter 5 (GLUT5), that absorbs fructose and most likely fructose released during the hydrolysis from the disaccharide sucrose [9,10,11]. More recently, other intestinal carbohydrate transporters have been implicated in glucose (GLUT2) and fructose (GLUT2, GLUT8, GLUT 12) absorption [12,13,14]. Because of the distinct transport routes for glucose and fructose, higher total intestinal carbohydrate absorption rates can be expected when glucose and fructose are co-ingested. In agreement, combined glucose and fructose ingestion has been shown to enhance intestinal carbohydrate absorption rates and results in higher exogenous carbohydrate oxidation rates during exercise compared with an equivalent amount of glucose [8,15,16].

Sucrose combines glucose and fructose monomers, and its hydrolysis is typically not rate-limiting for intestinal absorption [15,17]. In addition, recent work suggests that intact sucrose can also be transported as a disaccharide across the intestinal membrane [18]. Therefore, sucrose may represent an (even more) effective dietary source of fructose co-ingestion. In agreement, sucrose co-ingestion has been shown to further increase exogenous carbohydrate oxidation rates during exercise compared to glucose only [19,20]. However, sucrose co-ingestion during exercise does not seem to elevate exogenous carbohydrate oxidation rates beyond 1.2–1.3 g·min^−1^ [19,20], which is typically lower than 1.3–1.8 g·min^−1^ when fructose is co-ingested with glucose during exercise [8,16,21,22]. Exogenous carbohydrate oxidation rates do not appear to level off when increasing amounts of fructose are co-ingested [21]. In contrast, exogenous carbohydrate oxidation rates have been shown to plateau when moderate amounts of sucrose are co-ingested [19]. This may suggest that sucrose digestion and/or absorption becomes a limiting factor when large amounts of sucrose are co-ingested. Therefore, it remains unclear whether sucrose co-ingestion can be as effective as fructose co-ingestion to further augment exogenous carbohydrate oxidation rates when glucose ingestion is increased above 1.0–1.1 g·min^−1^.

We have recently shown that endurance-type exercise induces splanchnic hypoperfusion, resulting in a rapid increase in plasma I-FABP, a novel biomarker of intestinal damage [23]. Hypoperfusion-induced intestinal compromise may hamper athletic performance and can jeopardize early post-exercise recovery [24]. Meal ingestion and intestinal nutrient supply have the ability to increase the superior mesenteric artery blood flow and, hence, splanchnic perfusion [25,26]. Therefore, carbohydrate ingestion during endurance-type exercise may represent an effective nutritional strategy to attenuate splanchnic hypoperfusion and, as such, prevent exercise-induced gastrointestinal injury.

The present study assesses the impact of the combined ingestion of fructose or sucrose with glucose on exogenous carbohydrate oxidation rates. We hypothesized that both fructose and sucrose co-ingestion augment exogenous carbohydrate oxidation rates during exercise when compared to an isoenergetic amount of glucose. Furthermore, we hypothesized that fructose provided as part of the disaccharide sucrose is less effective as the same amount of fructose provided as a monosaccharide to further augment exogenous carbohydrate oxidation rates during exercise. We tested our hypothesis by subjecting 10 male cyclists to a 180 min exercise bout on four occasions, during which they ingested GLU (1.8 g·min^−1^ glucose), GLU + FRU (1.2 g·min^−1^ glucose + 0.6 g·min^−1^ fructose), GLU + SUC (0.6 g·min^−1^ glucose + 1.2 g·min^−1^ sucrose), or WAT (water placebo).

## 2. Materials and Methods

### 2.1. Subjects

Ten trained male cyclists or triathletes participated in this study (age: 26 ± 1 years, body weight: 74.8 ± 2.1 kg, body mass index: 21.5 ± 0.5 kg·m^−2^, maximal workload capacity (W_max_): 5.5 ± 0.1 W·kg^−1^, peak oxygen consumption (VO_2_peak): 65 ± 2 mL·kg^−1^·min^−1^). Subjects cycled at least 100 km·wk^−1^ and had a training history of >3 years. Subjects were fully informed on the nature and possible risks of the experimental procedures before their written informed consent was obtained. The study was approved by the Medical Ethical Committee of the Maastricht University Medical Centre, The Netherlands and conformed to standards for the use of human subjects in research outlined in the most recent version of the Helsinki Declaration. This trial was registered at clinicaltrials.gov as NCT0109617.

### 2.2. Pretesting

Baseline characteristics were determined during screening. Subject’s maximal workload capacity (W_max_) and peak oxygen consumption (VO_2_peak) were determined while performing a stepwise exercise test to exhaustion on an electronically braked cycle (Lode Excalibur, Groningen, The Netherlands), using an online gas-collection system (Omnical, Maastricht University, Maastricht, The Netherlands). After a 5 min warm up at 100 W, workload was set at 150 W and increased 50 W every 2.5 min until exhaustion. VO_2_peak was defined as the median of the highest consecutive values over 30 s. Maximal workload capacity was calculated as the workload in the last completed stage + workload relative to the time spent in the last incomplete stage: (time in seconds)/150 × 50 (W).

### 2.3. Diet and Activity before Testing

Subjects recorded their food intake and activity pattern 2 days before the first experimental exercise trial and followed the same diet and exercise activities prior to the other three trials. In addition, 5–7 days before each experimental testing day, subjects performed an intense exercise training session to deplete (^13^C-enriched) glycogen stores. Subjects were further instructed not to consume any food products with a high natural ^13^C abundance (carbohydrates derived from C_4_ plants: maize, sugar cane) at least 1 week before and during the entire experimental period to minimize any shift in background ^13^CO_2_ enrichment.

### 2.4. Experimental Design

Each subject performed four exercise trials which consisted of 180 min of cycling at 50% W_max_ while ingesting a glucose drink (GLU), an isoenergetic glucose + fructose drink (GLU + FRU), an isoenergetic glucose + sucrose drink (GLU + SUC), or plain water (WAT). To quantify exogenous carbohydrate oxidation rates, corn-derived glucose monohydrate (Cargill, Sas van Gent, The Netherlands), crystalline fructose and sugar cane-derived sucrose (Rafti Sugar Solutions BV, Wijchen, The Netherlands) were used, all of which have a high natural ^13^C abundance (−11.2, −11.4 and −11.2 δ‰ vs. Pee Dee Bellemnitella (PDB), respectively). The ^13^C enrichment of the ingested glucose, fructose, and sucrose were determined by gas chromatography-combustion-isotope ratio mass spectrometry (GC/C/IRMS; Agilent 7890A/GC5975C; MSD, Wilmington, DE, USA). To all drinks 20 mmol·L^−1^ of sodium chloride was added. The order of the experimental drinks was randomly assigned in a cross-over double-blinded design. Experimental trials were separated by 7–28 days.

### 2.5. Protocol

Subjects reported to the laboratory in the morning at 08:00 a.m. after an overnight fast (10 h) and having refrained from any strenuous activity or drinking any alcohol in the previous 24 h. On arrival in the laboratory, a Teflon catheter was inserted in an antecubital vein of an arm to allow repeated blood sampling during exercise. The subjects then mounted a cycle ergometer and a resting breath sample was collected in 10 mL Exetainer tubes (Labco Limited, Lampeter, UK), which were filled directly from a mixing chamber in duplicate to determine the ^13^C/^12^C ratio in the expired CO_2_. Next a resting blood sample (10 mL) was taken. Subjects then started a 180-min exercise bout at a work rate equivalent to 50% W_max_. Blood samples were collected at 30-min intervals throughout the 180 min exercise period. Expired breath samples were collected every 15 min until cessation of exercise. Measurements of oxygen consumption (VO_2_), carbon dioxide production (VCO_2_) and respiratory exchange ratio (RER) were obtained every 15 min for periods of 4 min through the use of a respiratory facemask, connected to an online gas-collection system [27].

During the first 3 min of exercise, subjects drank an initial bolus (600 mL) of one of the four experimental drinks: GLU, GLU + FRU, GLU + SUC, or WAT. Thereafter, every 15 min a beverage volume of a 150 mL was provided. The total fluid provided during the 180 min-exercise bout was 2.25 L. The GLU, GLU + FRU, and GLU + SUC drinks provided 1.8 g carbohydrate·min^−1^. The GLU drink provided 1.8 g·min^−1^ glucose, the GLU + FRU drink provided 1.2 g·min^−1^ glucose + 0.6 g·min^−1^ fructose, and the GLU + SUC drink provided 0.6 g·min^−1^ glucose + 1.14 g·min^−1^ sucrose. The amount of sucrose (1.14 vs. 1.2 g·min^−1^) was selected to allow exactly the same equimolar amounts of glucose and fructose provided in the GLU + SUC, GLU and GLU + FRU drinks.

Subjects were asked to rate their perceived exertion (RPE) every 30 min on a scale from 6 to 20 using the Borg category scale [28]. In addition, subjects were asked every 30 min to fill in questionnaire to rate possible gastrointestinal (GI) problems using a ten-point scale (1 = no complaints at all, 10 = very severe complaints). The questions consisted of six questions related to upper GI symptons (nausea, general stomach problems, belching, urge to vomit, heartburn, and stomach cramps), four questions related to lower GI complaints (flatulence, urge to defecate, intestinal cramps, and diarrhea), and four questions related to central or other symptoms (dizziness, headache, urge to urinate, and bloated feeling). All exercise tests were performed under normal and standard environmental conditions (18–22 °C dry bulb temperature and 55%–65% relative humidity). During the exercise trials, subjects were cooled with standing floor fans.

### 2.6. Analyses

Blood samples (10 mL) were collected in EDTA-containing tubes and centrifuged at 1000× *g* and 4 °C for 10 min. Aliquots of plasma were frozen in liquid nitrogen and stored at −80 °C until analysis. Plasma glucose and lactate were analyzed with a COBAS FARA semiautomatic analyzer (Roche). Plasma insulin concentrations were analyzed using commercially available kits (Elecsys Insulin assay, Roche, Ref: 12017547122; Mannheim, Germany). Plasma I-FABP levels were measured using an in-house developed enzyme-linked immunosorbent assay. The detection window of the I-FABP assay is 12.5–800 pg·mL^−1^, with an intra-assay and inter-assay coefficient of variation of 4.1% and 6.2%, respectively [23,29]. Breath samples were analyzed for ^13^C/^12^C ratio by gas chromatography continuous flow isotope ratio mass spectrometry (GC/C/IRMS; Finnigan, Bremen, Germany). From indirect calorimetry (VO_2_ and VCO_2_) and stable isotope measurements (breath ^13^CO_2_/^12^CO_2_ ratio), oxidation rates of total fat, total carbohydrate and exogenous carbohydrate were calculated.

### 2.7. Calculations

From VCO_2_ and VO_2_ (L·min^−1^), total carbohydrate and fat oxidation rates (g·min^−1^) were calculated using the stoichiometric equations of Frayn [30] with the assumption that protein oxidation during exercise was negligible:

(1) $$\mathrm{Carbohydrate}\;\mathrm{oxidation}=4.55\;{\mathrm{VCO}}_{2}-3.21\;{\mathrm{VO}}_{2}$$

(2) $$\mathrm{Fat}\;\mathrm{oxidation}=1.67\;{\mathrm{VO}}_{2}-1.67\;{\mathrm{VCO}}_{2}$$

The isotopic enrichment was expressed as δ per mil difference between the ^13^C/^12^C ratio of the sample and a known laboratory reference standard according to the formula of Craig [31]:

(3) $$\delta^{13}C=\left(\left(\frac{{}^{13}C/{}^{12}C\;\mathrm{sample}}{{}^{13}C/{}^{12}C\;\mathrm{standard}}\right)-1\right)\cdot 10^{3}$$

The δ^13^C was then related to an international standard (PDB-1). In the GLU, GLU + FRU, and GLU + SUC treatments, the rate of exogenous carbohydrate oxidation was calculated using the following [32]:

(4) $$\mathrm{Exogenous}\;\mathrm{glucose}\;\mathrm{oxidation}={\mathrm{VCO}}_{2}\cdot \;\left(\frac{\delta \;\mathrm{Exp}-{\delta \;\mathrm{Exp}}_{\mathrm{bkg}}}{\delta \;\mathrm{Ing}-{\delta \;\mathrm{Exp}}_{\mathrm{bkg}}}\right)\left(\frac{1}{k}\right)$$

in which δ Exp is the ^13^C enrichment of expired air during exercise at different time points, δ Ing is the ^13^C enrichment of the ingested carbohydrate solution, δ Exp_bkg_ is the ^13^C enrichment of expired air in the WAT treatment (background) at different time points and *k* is the amount of CO_2_ (in L) produced by the oxidation of 1 g of glucose (*k* = 0.7467 L of CO_2_·g^−1^ of glucose).

A methodological consideration when using ^13^CO_2_ in expired air to calculate exogenous substrate oxidation is the trapping of ^13^CO_2_ in the bicarbonate pool, in which an amount of CO_2_ arising from decarboxylation of energy substrates is temporarily trapped [33]. However, during exercise the CO_2_ production increases several-fold so that a physiological steady state condition will occur relatively rapidly, and ^13^CO_2_ in the expired air will be equilibrated with the ^13^CO_2_/H^13^CO_3_^−^ pool, respectively. Recovery of the ^13^CO_2_ from oxidation will approach 100% after 60 min of exercise when dilution in the bicarbonate pool becomes negligible [33,34]. As a consequence of this, calculations on substrate oxidation were performed over the last 120 min of exercise (60–180 min).

### 2.8. Statistical Analyses

Plasma and substrate utilization parameters are expressed as means ± SEM, RPE and GI distress scores are expressed as median and interquartile range. A sample size of 10 was calculated with a power of 80% and an alpha level of 0.05 to detect a ~20% difference in exogenous carbohydrate oxidation between treatments [20]. For all data, the normality of the distribution was confirmed after visual inspection and the use of Shapiro-Wilk tests. A one-way repeated measures ANOVA with treatment as factor was used to compare differences in substrate utilization parameters between treatments. In case of significant *F*-ratios, Bonferroni post-hoc tests were applied to locate the differences. A two-way repeated measures ANOVA with time and treatment as factors was used to compare differences in plasma parameters between treatments and over time. In case of significant *F*-ratios, paired *t*-tests were used to locate the differences. A Friedman test was performed to compare RPE and GI distress scores between treatments. In case of significant χ^2^, post hoc analysis with Wilcoxon signed-rank test was conducted. Data evaluation was performed using SPSS (version 21.0, IBM Corp., Armonk, NY, USA). Statistical significance was set at *p* < 0.05.

## 3. Results

### 3.1. Indirect Calorimetry

Data for VO_2_, RER, and total carbohydrate and fat oxidation rates over the 60 to 180 min exercise period are presented in Table S1. VO_2_ did not differ between the four experimental treatments (*p* = 0.301). RER in WAT was lower compared with GLU + FRU and GLU + SUC (*p* < 0.05). Total carbohydrate oxidation rates were lower in WAT compared with GLU + FRU and GLU + SUC treatments (*p* < 0.05). No significant differences in total carbohydrate oxidation rates were observed between GLU, GLU + FRU and GLU + SUC (pairwise comparisons: all *p* ≥ 0.172). Total fat oxidation rates were higher in WAT compared to GLU + SUC (*p* = 0.010). No significant differences in total fat oxidation rates were observed between GLU, GLU + FRU and GLU + SUC (pairwise comparisons: all *p* ≥ 0.443).

### 3.2. Stable-Isotope Measurements

**Figure 1:**
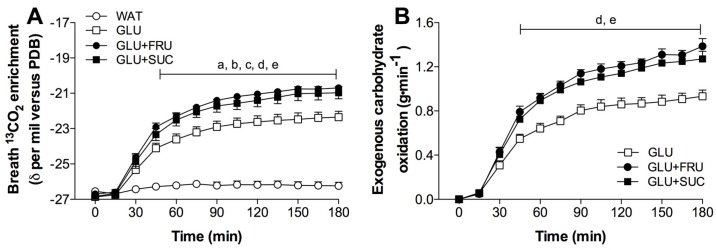
Breath ^13^CO_2_ enrichments (**A**) and exogenous carbohydrate oxidation rates (**B**) during exercise without ingestion of carbohydrate (WAT), with the ingestion of glucose (GLU), with the ingestion of glucose and fructose (GLU + FRU), or with the ingestion of glucose and sucrose (GLU + SUC). Data were analsysed with a two-way repeated measures ANOVA (time-treatment). Data are presented as means ± SEM. *N* = 10. a, denotes GLU significantly different from WAT; b, denotes GLU + FRU significantly different from WAT; c, denotes GLU + SUC significantly different from WAT; d, denotes GLU + FRU significantly different from GLU; e, denotes GLU + SUC significantly different from GLU (*p* < 0.05).

Changes in isotopic composition of expired CO_2_ in response to exercise with ingestion of GLU, GLU + FRU, GLU + SUC or WAT are presented in Figure 1A. Resting breath ^13^CO_2_ enrichments did not differ between treatments, and averaged −26.55 ± 0.13, −26.86 ± 0.16, −26.69 ± 0.14, −26.83 ± 0.18 δ‰ versus PDB for WAT, GLU, GLU + FRU, and GLU + SUC, respectively. No significant increases in expired breath ^13^CO_2_ enrichments were observed in the water only treatment (WAT; *p* = 0.096). In contrast, expired breath ^13^CO_2_ enrichments strongly increased to up to −22.36 ± 0.33, −20.70 ± 0.18, and −20.97 ± 0.34 δ‰ versus PDB in the GLU, GLU + FRU, and GLU + SUC treatments, respectively (time x treatment, *p* < 0.001). The slight shift in expired breath ^13^CO_2_ enrichments in the WAT treatment was used as a background correction for the calculation of exogenous carbohydrate oxidation rates in the GLU, GLU + FRU and GLU + SUC treatments.

### 3.3. Exogenous and Endogenous Carbohydrate Oxidation Rates

**Figure 2:**
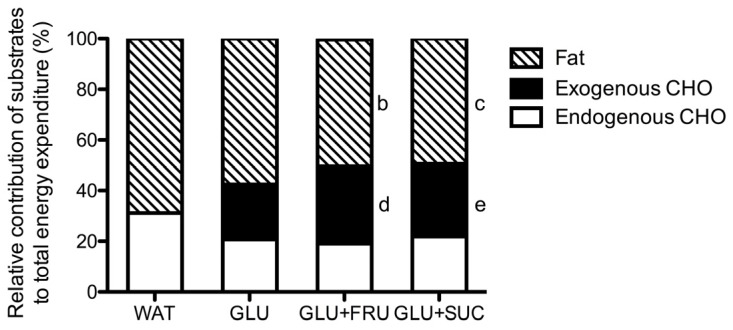
Relative contribution of substrates to total energy expenditure calculated for the 60- to 180 min period of exercise without the ingestion of carbohydrate (WAT), with the ingestion of glucose (GLU), with the ingestion of glucose and fructose (GLU + FRU), or with the ingestion of glucose and sucrose (GLU + SUC). Data were analsysed with a repeated measures ANOVA (treatment). Data are presented as means ± SEM. *N* = 10; b, denotes GLU + FRU significantly different from WAT; c, denotes GLU + SUC significantly different from WAT; d, denotes GLU + FRU significantly different from GLU; e, denotes GLU + SUC significantly different from GLU (*p* < 0.05).

In the GLU, GLU + FRU, and GLU + SUC treatments, the calculated exogenous carbohydrate oxidation rates increased significantly over time (Figure 1B, *p* < 0.001). Peak exogenous carbohydrate oxidation rates were 51% ± 9% and 40% ± 12% higher in GLU + FRU and GLU + SUC when compared to GLU (1.40 ± 0.06 and 1.29 ± 0.07 vs. 0.96 ± 0.06 g·min^−1^, respectively: *p* < 0.05). Peak exogenous carbohydrate oxidation rates did not differ between GLU + FRU and GLU + SUC (*p* = 0.999). Assessed over the last 120 min of exercise, average exogenous carbohydrate oxidation rates were higher in the GLU + FRU and GLU + SUC treatments compared to the GLU treatments (1.19 ± 0.12, 1.13 ± 0.21, and 0.82 ± 0.16 g·min^−1^, respectively: *p* < 0.05). No differences were observed in exogenous carbohydrate oxidation rates between GLU + FRU and GLU + SUC (*p* = 0.999). No significant differences in endogenous carbohydrate oxidation rates were observed between treatments (*p* = 0.112). The relative contribution of substrates to total energy expenditure during exercise is presented in Figure 2. 

### 3.4. Plasma Metabolites

**Figure 3:**
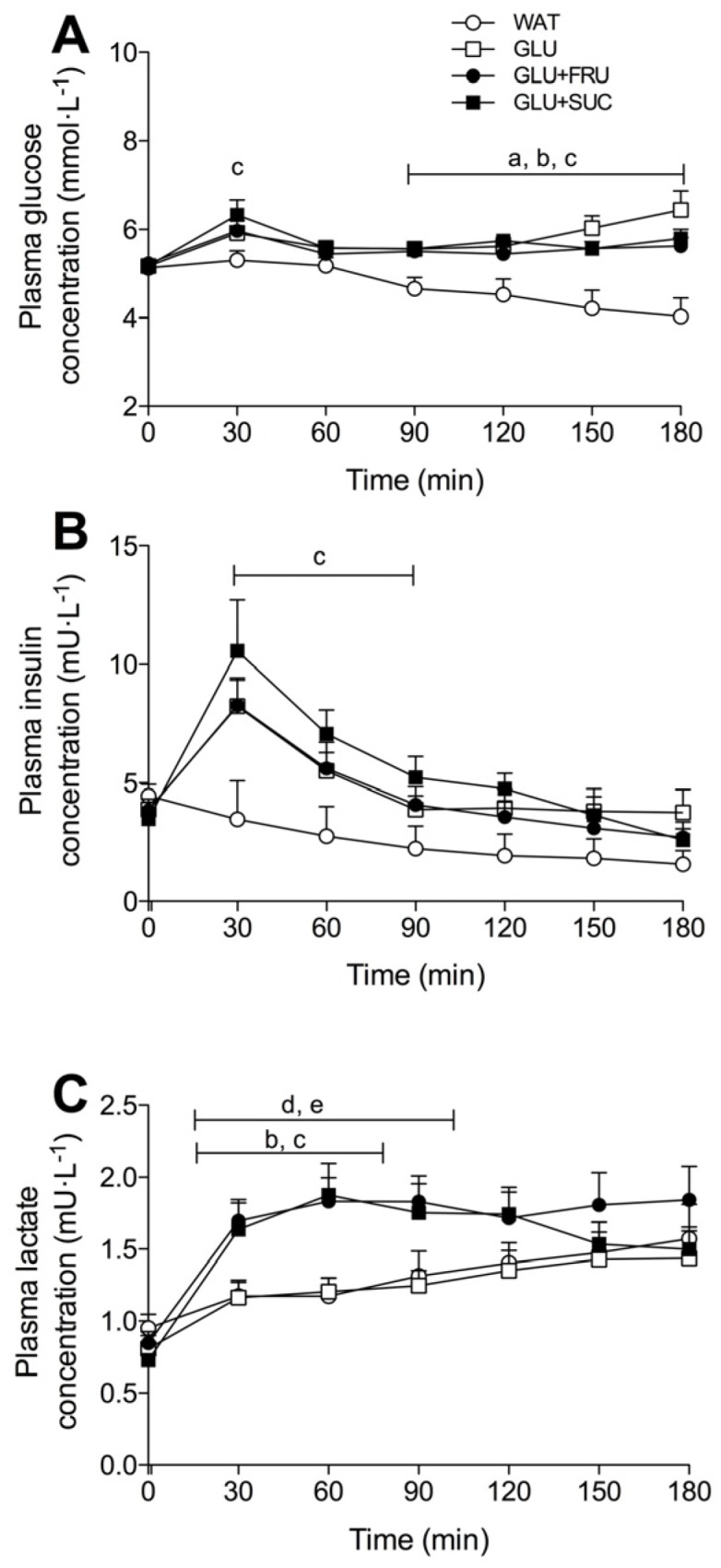
Plasma glucose (**A**), insulin (**B**), and lactate (**C**) concentrations during exercise without ingestion of carbohydrate (WAT), with the ingestion of glucose (GLU), with the ingestion of glucose and fructose (GLU + FRU), or with the ingestion of glucose and sucrose (GLU + SUC). Data were analsysed with a two-way repeated measures ANOVA (time-treatment). Data are presented as means ± SEM. *N* = 10; a, denotes GLU significantly different from WAT; b, denotes GLU + FRU significantly different from WAT; c, denotes GLU + SUC significantly different from WAT; d, denotes GLU + FRU significantly different from GLU; e, denotes GLU + SUC significantly different from GLU (*p* < 0.05).

Plasma glucose, insulin, and lactate concentrations are shown in Figure 3. Plasma glucose concentrations showed a transient increase at *t* = 30 min in the GLU, GLU + FRU and GLU + SUC treatments, but plasma glucose concentrations at *t* = 30 min were only significantly higher in GLU + SUC compared to WAT (*p* = 0.028). Plasma glucose concentrations decreased in the WAT treatment compared to the GLU, GLU + FRU and GLU + SUC treatments (time x treatment interaction: *p* < 0.001). Plasma glucose concentrations were significantly lower in the WAT treatment compared to the GLU, GLU + FRU and GLU + SUC treatments from *t* = 90 min onwards (*p* < 0.05). Plasma insulin concentrations increased in the GLU, GLU + FRU and GLU + SUC treatments, peaking at *t* = 30 after glucose ingestion (8.2 ± 1.2, 8.3 ± 1.1, and 10.6 ± 2.2 mU·L^−1^, respectively), and then declined throughout exercise. In contrast, plasma insulin concentrations declined throughout the entire exercise bout in the WAT treatment (time x treatment interaction: *p* < 0.001).

**Figure 4:**
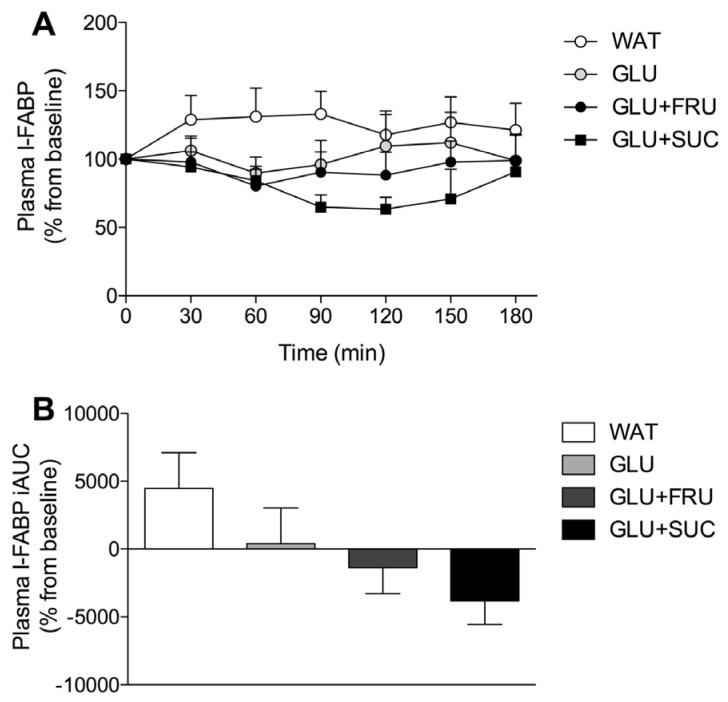
Plasma I-FABP concentrations during exercise (**A**) and (area under the curve (AUC) of percentage I-FABP change during exercise (**B**) without ingestion of carbohydrate (WAT), with the ingestion of glucose (GLU), with the ingestion of glucose and fructose (GLU + FRU), or with the ingestion of glucose and sucrose (GLU + SUC). Plasma I-FABP (A) was analsysed with a two-way repeated measures ANOVA (time-treatment). Plasma I-FABP iAUC was analysed with a repeated measures ANOVA (treatment). Data are presented as means ± SEM. *N* = 10. Differences between treatments did not reach statistical significance (*p* > 0.05).

Plasma lactate concentrations increased in all treatments, but this increase was much greater in the GLU + FRU and GLU + SUC treatments when compared with the WAT and GLU treatments (time x treatment interaction: *p* < 0.001). Plasma I-FABP levels, depicted as a percentage change from individual baseline values, did not change significantly over time (*p* = 0.764; Figure 4A). Area under the curve (AUC) calculations of the percentage change from individual I-FABP baseline values did not differ between treatments (*p* = 0.101; Figure 4B).

### 3.5. Gastrointestinal Distress and Rating of Percieved Exertion

Total upper GI distress scores were 62 (53–83), 109 (67–147), 69 (57–96) and 74 (53–79), in the WAT, GLU, GLU + FRU, GLU + SUC treatments, respectively, and were significantly higher in the GLU compared to the WAT, GLU + FRU, and GLU + SUC treatments (*p* < 0.05). Low GI distress scores were observed for other symptons, with the exception of urge to urinate which did not differ between treatments (*p* = 0.455). Ratings of perceived exertion did not differ between treatments and averaged 13 (11–14), 14 (13–14), 13 (12–13), and 13 (12–14) for WAT, GLU, GLU + FRU, and GLU + SUC, respectively (*p* = 0.056).

## 4. Discussion

The present study shows that the combined ingestion of glucose and fructose (1.2 g·min^−1^ glucose plus 0.6 g·kg^−1^ fructose or 0.6 g·min^−1^ glucose plus 1.2 g·min^−1^ sucrose) further increases exogenous carbohydrate oxidation rates compared to the ingestion of an isocaloric amount of glucose only. Furthermore, combined ingestion of glucose plus fructose or sucrose resulted in less GI complaints when compared with the ingestion of glucose only.

Previous work suggests that exogenous glucose oxidation rates are limited by intestinal glucose absorption [5,7]. Because fructose is absorbed through a different intestinal transport route, higher total intestinal carbohydrate absorption rates can be expected when glucose and fructose are co-ingested. Therefore, we hypothesized that fructose co-ingestion with glucose would increase total carbohydrate oxidation rates during prolonged exercise. Furthermore, we hypothesized that fructose provided as part of the disaccharide sucrose is less effective as the same amount of fructose provided as a monosaccharide to further augment exogenous glucose oxidation rates during exercise.

In the present study, we observed that the ingestion of an ample amount of glucose only results in peak exogenous carbohydrate oxidation rates of 0.96 ± 0.06 g·min^−1^ (Figure 1). These rates are in line with previous work and confirm that exogenous glucose oxidation rates will not rise above 1.0–1.1 g·min^−1^ when only glucose is ingested during exercise [7,16,22,35,36]. Combined ingestion of glucose plus fructose or glucose plus sucrose in the present study resulted in peak exogenous carbohydrate oxidation rates of 1.40 ± 0.06 and 1.29 ± 0.07 g·min^−1^, respectively (Figure 1). These data confirm previous observations showing 35%–55% higher exogenous carbohydrate oxidation rates following fructose co-ingestion when compared to the ingestion of glucose only during exercise [8,16]. While sucrose co-ingestion has also been shown to increase exogenous carbohydrate oxidation rates [19,20], maximal exogenous carbohydrate oxidation rates appear lower when sucrose [19,20] as opposed to fructose [8,16,21,22] is co-ingested during exercise (1.2–1.3 vs. 1.3–1.8 g·min^−1^, respectively). In the present study, we compared the impact of equimolar amounts of fructose co-ingestion provided as its monosaccharide or provided as sucrose on exogenous carbohydrate oxidation rates during exercise in the same cohort of athletes. We extend on previous work by showing no significant differences in peak exogenous carbohydrate oxidation rates between the GLU + FRU and GLU + SUC treatments (*p* = 0.999). These results suggest that sucrose intestinal digestion and/or absorption are not rate-limiting for subsequent oxidation. Consequently, we demonstrate that fructose co-ingestion provided as part of the disaccharide sucrose does not differ from an equivalent amount of fructose provided as a monosaccharide to augment exogenous carbohydrate oxidation rates during endurance type exercise. 

The ingestion of glucose only resulted in substantially higher GI distress when compared to the WAT, GLU + FRU and GLU + SUC treatments (*p* < 0.05). The lower total GI distress with fructose or sucrose co-ingestion seems to suggest that these treatments result in less carbohydrate accumulation in the GI tract, possibly caused by more rapid intestinal carbohydrate absorption when compared with the ingestion of glucose only [37]. To further evaluate potential underlying mechanisms of the GI discomfort, we also measured plasma I-FABP as a marker of intestinal damage. Previous work has shown that exercise resulted in a rapid appearance of this marker in blood, which correlated with splanchnic hypoperfusion [23]. Though not significant, we observed lower I-FABP release in the GLU, GLU + FRU, and GLU + SUC groups compared with the WAT treatment (*p* = 0.101, Figure 4). The observed increase in plasma I-FABP in the WAT treatment was lower when compared to our previous work [29]. This may be explained by differences in the exercise protocols. In our previous work, subjects cycled for 60 min at 70% W_max_ vs. 180 min at 50% W_max_ in the current study. This suggests that exercise intensity may be a more important modulator of peak plasma I-FABP levels than exercise duration. It remains to be established whether carbohydrate ingestion may reduce exercise-induced GI compromise during higher intensity exercise.

After ingestion and intestinal absorption, fructose is metabolized in the liver and subsequently released in the systemic circulation as lactate or converted to glucose via gluconeogenesis, which is mainly released or used for liver glycogen synthesis depending on the need to maintain plasma glucose levels [38]. We observed no significant differences in plasma glucose concentrations between the GLU, GLU + FRU, and the GLU + SUC treatments (Figure 3). We have recently shown that sucrose ingestion does not preserve liver glycogen concentrations more than glucose ingestion during exercise [39]. Therefore, it seems unlikely that hepatic glycogenesis was a major fate of the ingested fructose during exercise. We did observe elevated plasma lactate concentrations in the GLU + FRU and GLU + SUC treatments when compared to the GLU treatment. Fructose co-ingestion has been shown to increase plasma lactate production and oxidation, with a minimal amount of fructose being directly oxidized [40]. Therefore, fructose or sucrose co-ingested with glucose appears to be effectively absorbed in the intestine and transported to the liver where it is metabolized to lactate, released in the circulation and subsequently oxidized.

Exogenous glucose oxidation rates have been shown to correlate with exercise performance during prolonged, moderate- to high-intensity exercise [2]. Fructose co-ingestion further improves exogenous carbohydrate oxidation rates and has shown to improve exercise performance compared to an isocaloric amount of glucose [8,14,16,22,41,42,43]. The latter has been attributed to a combination of higher exogenous carbohydrate oxidation rates and decreased GI distress [41,43]. Although we did not assess exercise performance, we observed increased exogenous carbohydrate rates and lower GI distress following fructose co-ingestion. Furthermore, exogenous carbohydrate oxidation rates and GI distress levels did not differ between the GLU + FRU and GLU + SUC treatments. Therefore, our data suggest that both fructose and sucrose represents proper ingredients for sports drinks to further increase exogenous carbohydrate oxidation rates during exercise.

This study presents several limitations. First, we assessed whole-body exogenous carbohydrate oxidation rates following fructose co-ingestion, which does not provide insight in the specific site of oxidation. The increased exogenous carbohydrate oxidation rates following fructose co-ingestion is likely largely attributed to increased lactate oxidation in muscle [40,44]. However, hepatic fructose conversion into glucose and/or lactate costs energy [45], thereby decreasing energy efficiency and possibly increasing hepatic carbohydrate oxidation rates. Therefore, the observed 46% ± 8% higher exogenous carbohydrate oxidation rates following fructose co-ingestion may slightly overestimate increased energy availability and exogenous carbohydrate oxidation rates in muscle. Secondly, we cannot exclude the possibility that sucrose co-ingestion is less effective at increasing exogenous carbohydrate oxidation when compared to fructose monosaccharide co-ingestion when total carbohydrate ingestion rates are higher than provided in the current study. It has been suggested that sucrose digestion and/or absorption becomes a limiting factor to further increase exogenous carbohydrate oxidation rates when total carbohydrate intakes levels exceed 1.8 g·min^−1^ [19]. However, such carbohydrate ingestion rates may be impractically high and result in GI distress that may be detrimental to exercise performance [43]. Therefore, we provided total carbohydrate ingestion rates that are more practical and have shown to increase performance [42].

## 5. Conclusions

We conclude that fructose co-ingestion provided either as a monosaccharide or as sucrose strongly increases exogenous carbohydrate oxidation rates during prolonged exercise in trained cyclists. When ingesting large amounts of carbohydrates during exercise, co-ingestion of fructose or sucrose will lower GI distress and increase the capacity for exogenous carbohydrate oxidation.

## Supplementary Materials

The following are available online at http://www.mdpi.com/2072-6643/9/2/167/s1, Table S1: Oxygen uptake (VO_2_), respiratory exchange ratio (RER), total carbohydrate (CHO) oxidation (CHOtot), total fat oxidation (FATtot), endogenous carbohydrate (Endogenous CHO), exogenous carbohydrate (Exogenous CHO) oxidation and peak exogenous carbohydrate oxidation (peak exogenous CHO) rates during cycling exercise with ingestion of GLU, GLU + FRU, and GLU + SUC, and WAT.
