# Supplementary material for: Fructose and Sucrose Intake Increase Exogenous Carbohydrate Oxidation during Exercise
Source: Nutrients. 2017 Feb 20;9(2):167. doi: 10.3390/nu9020167 (PMC5331598; doi:10.3390/nu9020167)
Supplement: Supplementary file 1 [file nutrients-09-00167-s001.docx]

| **Supplemental Table 1.** Oxygen uptake (VO_2_), respiratory exchange ratio (RER), total carbohydrate (CHO) oxidation (CHOtot), total fat oxidation (FATtot), endogenous carbohydrate (Endogenous CHO), and exogenous carbohydrate (Exogenous CHO) oxidation during cycling exercise with ingestion of GLU, GLU+FRU, and GLU+SUC, and WAT. | | | | | | | | |
| --- | --- | --- | --- | --- | --- | --- | --- | --- |
|  | **Time** | **VO2**  **(L**·**min^-1^)** | **RER** | **CHOtot**  **(g**·**min^-1^)** | **FATtot**  **(g**·**min^-1^)** | **Endogenous CHO**  **(g**·**min^-1^)** | **Exogenous**  **CHO**  **(g**·**min^-1^)** | **Peak Exogenous CHO**  **(g**·**min^-1^)** |
| **WAT** | 60-90 | 2.94±0.06 | 0.81±0.01 | 1.35±0.18 | 0.95±0.09 | 1.35±0.18 |  |  |
|  | 90-120 | 2.96±0.06 | 0.80±0.01 | 1.19±0.19 | 1.02±0.09 | 1.19±0.19 |  |  |
|  | 120-150 | 3.00±0.06 | 0.79±0.04 | 1.07±0.18 | 1.08±0.09 | 1.07±0.18 |  |  |
|  | 150-180 | 3.04±0.06 | 0.78±0.05 | 1.01±0.60 | 1.12±0.09 | 1.01±0.19 |  |  |
| **GLU** | 60-90 | 2.96±0.06 | 0.83±0.01 | 1.63±0.15 | 0.86±0.07 | 0.91±0.13 | 0.72±0.05 |  |
|  | 90-120 | 2.99±0.06 | 0.83±0.01 | 1.65±0.15 | 0.86±0.07 | 0.82±0.12 | 0.83±0.06 | 0.96±0.06 |
|  | 120-150 | 2.97±0.06 | 0.82±0.01 | 1.58±0.14 | 0.88±0.07 | 0.72±0.10 | 0.85±0.06 |  |
|  | 150-180 | 3.00±0.06 | 0.82±0.01 | 1.55±0.14 | 0.90±0.07 | 0.65±0.11 | 0.89±0.06 |  |
| **GLU+FRU** | 60-90 | 3.00±0.06 | 0.85±0.01 | 1.96±0.10 | 0.76±0.04 | 0.93±0.11 | 1.03±0.04^b^ |  |
|  | 90-120 | 3.01±0.05 | 0.85±0.01 | 1.94±0.12 | 0.77±0.05 | 0.76±0.12 | 1.18±0.04^b^ | 1.40±0.06 |
|  | 120-150 | 3.05±0.05 | 0.84±0.01^a^ | 1.87±0.12^a^ | 0.81±0.04 | 0.61±0.11 | 1.25±0.04^b^ |  |
|  | 150-180 | 3.12±0.06 | 0.84±0.01^a^ | 1.96±0.15^a^ | 0.80±0.05^a^ | 0.63±0.14 | 1.33±0.05^b^ |  |
| **GLU+SUC** | 60-90 | 2.90±0.08 | 0.87±0.02^a^ | 2.08±0.18^a^ | 0.67±0.09^a^ | 1.09±0.17 | 0.98±0.06^b^ |  |
|  | 90-120 | 2.95±0.08 | 0.85±0.01^a^ | 1.95±0.15^a^ | 0.73±0.08^a^ | 0.85±0.14 | 1.10±0.07^b^ | 1.29±0.07 |
|  | 120-150 | 2.98±0.08 | 0.85±0.01^a^ | 1.91±0.14^a^ | 0.77±0.07^a^ | 0.72±0.12 | 1.19±0.07^b^ |  |
|  | 150-180 | 3.00±0.08 | 0.85±0.01^a^ | 1.93±0.15^a^ | 0.77±0.07^a^ | 0.68±0.12 | 1.25±0.07^b^ |  |
| WAT: ingestion of water only; GLU: ingestion of glucose; GLU+FRU: ingestion of glucose and fructose; GLU+SUC: ingestion of glucose and sucrose. Data are presented as means ±SE. *N*=10. ^a^, denotes significantly different from WAT; ^b^, denotes significantly different from GLU *(P* < 0.05). | | | | | | | |  |
